# Low Avidity T Cells Do Not Hinder High Avidity T Cell Responses Against Melanoma

**DOI:** 10.3389/fimmu.2019.02115

**Published:** 2019-09-06

**Authors:** Kalliopi Ioannidou, Olivier Randin, Aikaterini Semilietof, Hélène Maby-El Hajjami, Petra Baumgaertner, Dominique Vanhecke, Daniel E. Speiser

**Affiliations:** Department of Oncology, Lausanne University Hospital Center (CHUV), Lausanne, Switzerland

**Keywords:** avidity, T cell response, melanoma, IFN-γ Elispot assay, cytotoxicity assay, adoptive T cell transfer

## Abstract

The efficacy of T cells depends on their functional avidity, i. e., the strength of T cell interaction with cells presenting cognate antigen. The overall T cell response is composed of multiple T cell clonotypes, involving different T cell receptors and variable levels of functional avidity. Recently, it has been proposed that the presence of low avidity tumor antigen-specific CD8 T cells hinder their high avidity counterparts to protect from tumor growth. Here we analyzed human cytotoxic CD8 T cells specific for the melanoma antigen Melan-A/MART-1. We found that the presence of low avidity T cells did not result in reduced cytotoxicity of tumor cells, nor reduced cytokine production, by high avidity T cells. *In vivo* in NSG-HLA-A2 mice, the anti-tumor effect of high avidity T cells was similar in presence or absence of low avidity T cells. These data indicate that low avidity T cells are not hindering anti-tumor T cell responses, a finding that is reassuring because low avidity T cells are an integrated part of natural T cell responses.

## Introduction

The strength of the interaction between T cells and antigen-bearing cells depends on the TCR affinity to peptide-MHC, and on multiple co-receptors that together determine the overall binding kinetic and consequent signaling into T cells. The multiple receptor-ligand pairs are organized in the highly complex immune synapse. Many studies have provided great insight into the structure and function of the synapse (1, 2). However, structural data are not sufficient to calculate or predict the function outcome. Therefore, cellular functional assays are still of central importance for assessing the strength that is to say the “functional avidity,” of T cell interactions and their functional consequence *in vitro* and *in vivo*, including the net overall outcome of the T cell response (3, 4). Standard methods to determine the functionality avidity (hereafter abbreviated with “avidity”) are cytotoxicity or IFN-γ Elispot assays with titrated concentrations of antigenic peptide. The peptide concentration that mediates half-maximal activity is called EC_50_ and used as avidity measure (5, 6).

High and low avidity T cells seem to participate both in immune responses against tumors in humans and animals. There is a consensus that higher avidity T cells contribute more strongly to immunity as opposed to lower avidity T cells (7–10). Whether or not they act synergistically is less well-known. It has been reported that chronic antigen exposure may tolerize T cells whereby the degree of tolerization may differ depending on the T cell's avidity (11, 12).

It is challenging to determine the net contribution of individual T cell clonotypes to immunity (i.e., to the disease outcome). The best experimental method consists of the transfer of defined T cell populations to individuals bearing an infection or a tumor, and to quantify the consequent immune protection. This procedure is straightforward for murine studies. For humans the possibilities are limited. However, adoptive T cell therapy (ACT) is increasingly performed, for example for the treatment of patients with metastatic melanoma. This technique relies on the isolation of autologous tumor-infiltrating lymphocytes (TILs) from tumor biopsies or CD8 T cell clones derived from peripheral blood T cells that are expanded and re-injected into patients, with the aim that these TILs/CD8 T cells then directly kill tumor cells (13, 14). Even though technically and clinically challenging, TIL-ACT showed promising results in terms of objective clinical responses and durability of responses (15–17).

Despite these clinical successes, ACT has limitations in availability and generation of therapeutic tumor-reactive T cells of sufficiently high avidity for a larger group of patients. Traditionally, generating or selecting high avidity TILs *in vitro* include MHC/antigen tetramer staining and sorting, with stronger tetramer binding indicative of higher avidity and tumor reactivity (8, 18). Alternatively, T cells can also be expanded *in vitro* in the presence of low concentrations of peptide, which selects for T cells with higher avidity and greater tumor reactivity (19). Nevertheless, its widespread application is hindered by the laborious nature and limited success rate of isolating and expanding TILs for patient treatment. To overcome this disadvantage, peripheral T cells can be genetically engineered to express TCRs or chimeric antigen receptors with a high avidity and excellent specificity for target antigen, as well as costimulatory molecules that provide the T cells with enhanced properties required for effective ACT therapy (20, 21). Several studies have shown measurable success of genetically modified T cells in melanoma patients (22, 23), but they also demonstrated the occurrence of unexpected toxicities.

Considering the co-existence of high and low avidity T cells within tumors, we thought it is worthwhile to determine whether the presence of low avidity T cells in our experimental systems *in vitro* and *in vivo* would hinder the high avidity T cells in their activities against melanoma. Therefore, we compared the T cell's function in experiments using T cell clones with defined avidity, by using them individually and in parallel to mixtures of T cell cones with different avidities.

## Results

### Similar Killing of Melanoma Cells by High Avidity Cytotoxic T Cells in Presence or Absence of Low Avidity T Cells

**Figure 1:**
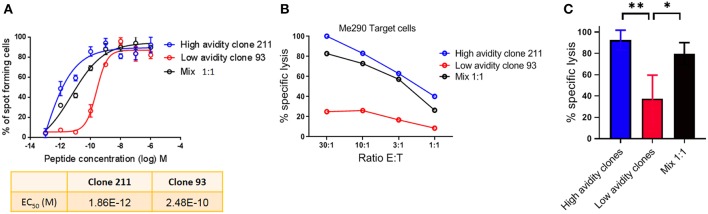
Cytotoxicity by T cell clones, alone and in mixed cultures of high and low avidity clones. **(A)** Peptide (Melan-A peptide EAAGIGILTV; “EAA”), titration curves in an IFN-γ Elispot assay, to determine the functional avidity of the clones used for the subsequent killing assays. **(B)** The low avidity clone 93 did not inhibit the lysis of melanoma cells by the high avidity clone 211. **(C)** Average ± standard deviation, and statistical comparisons (One-way Anova) of four independent cytotoxicity assays at the E:T ratio of 30:1, using the clones described in Table 1. *P*-values **<0.01; *<0.05. No peptide was added in **(B,C)**; tumor cell recognition was based on endogenous expression of Melan-A by the Me290 melanoma target cells. Ratio E:T; effector to target cell ratio.

For the analysis of human CD8 T cell responses with different avidities, we generated T cell clones from HLA-A^*^02:01 melanoma patients and determined their functional avidity (Figure 1A and Table 1) as described previously (3, 24). Subsequently, we determined the cytotoxicity and found that the low avidity clones showed lower killing of Me290 melanoma cells as compared to the high avidity clones (Figure 1B). Then we used these clones to ask the question whether the low avidity clone could influence the function of the more efficient clones. We found that the presence of the low avidity T cell clone 93 did not hinder the cytotoxicity of the high avidity clone 211 when they were mixed together (Figure 1B). The killing by the mixed T cells was slightly lower which may have been due to the fact that these wells contained only half the number of the high affinity clone than in the conditions with only a single clone, since the remaining cells of the mix were the low avidity T cells. The compiled data from four independent experiments show that the differences were statistically significant, i.e., that the low avidity clones indeed exerted weaker killing as compared to the mixed clones as in comparison to the high avidity clones (Figure 1C).

**Table 1:** Overview of the CD8 T cell clones used in this study.

**Clone**	**Patient (Lau)**	**Functional avidity group**	**Elispot EC_**50**_ (M)**	**R square**
201	975	High	1.80E-12	0.9533
211	1,013	High	1.86E-12	0.9428
212	1,013	High	7.13E-11	0.9687
214	1,013	High	2.71E-10	0.9708
93	944	Low	2.48E-10	0.9840
95	944	Low	2.0E-10	N/A
33	618	Low	1.28E-09	0.9643
35	618	Low	5.49E-09	0.9455

*All clones are specific for Melan-A/HLA-A2. They were generated from patient's PBMC as described in Material and Methods. IFN-γ Elispot assays with titrated Melan-A peptide were performed to determine the peptide concentration at half maximal activity (EC_50_), reflecting the functional avidity. Low avidity clones are considered the ones having an EC_50_ above 2.50E-10 (M). The R square values represent the goodness of sigmoid curve fit. As an exception, the functional avidity of clone 95 was determined using a cytotoxicity assay, in contrast to all other clones for which the functional avidity was determined by IFN-γ Elispot assays. We have previously shown that the two assays generate similar results (3)*.

### Pre-incubation of Melanoma Cells With Low Avidity T Cells Does Not Reduce Their Susceptibility to Lysis

**Figure 2:**
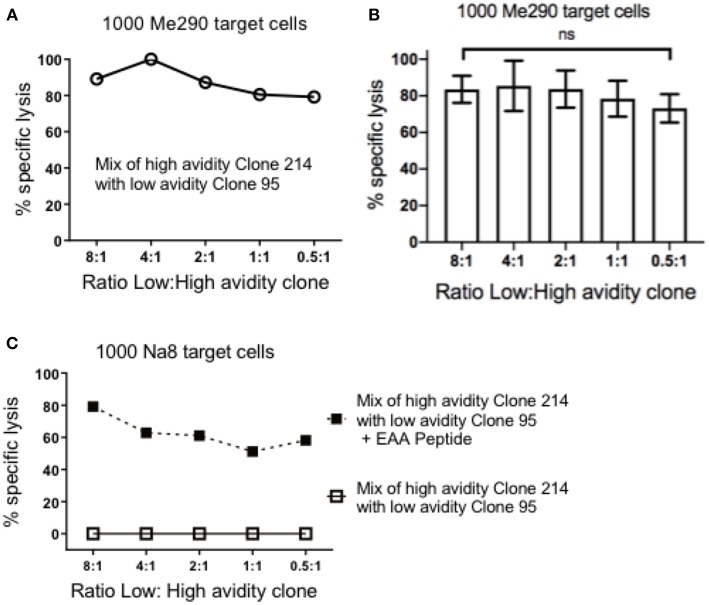
Pre-incubation of target cells with the low avidity T cell clones did not inhibit the cytotoxicity of the high avidity T cell clones. **(A)** At the constant concentration of 1,000 target cells in every well, different numbers of low avidity T cells (clone 95) were pre-incubated for 1.5 h with Me290 melanoma target cells (without synthetic peptide). Subsequently, the high avidity T cells (clone 214) was added in the following cell numbers (ratios) of low avidity clone: high avidity clone, at 80.000:10.000 (8:1), 40.000:10.000 (4:1), 20.000:10.000 (2:1), 10.000:10.000 (1:1), 5.000:10.000 (0.5:1), and incubated for further 3 h to determine specific lysis as described in the methods section. **(B)** Average ± standard deviation of three independent assays. No statistically significant differences were detected (One-way Anova). **(C)** Control experiment with the Melan-A negative melanoma cell line Na8, with and without the addition of synthetic Melan-A peptide (“EAA”; EAAGIGILTV) at the concentration of 1 μM, showing results that are representative for two experiments.

Next, we assessed if pre-incubation of the low avidity T cells could reduce the subsequent susceptibility of target cells to high avidity cytotoxic T cells. The concentration of the target cells was kept steady at 1,000 cells per plate, and the mix ratio of low: high avidity T cells was gradually increased. Figure 2A shows data of an experiment in which we observed strong specific lysis of Me290 target cells even at the high concentration of a low avidity T cell clone (ratio 8:1 of low:high avidity clones). In all the concentrations of the low avidity clone, the differences remained small, with overall high cytotoxicity in all conditions. Figure 2B presents the compiled data of three independent experiments, revealing similar results with only small differences (statistically not significant) between the conditions with different ratios of low and high avidity clones. In parallel, the Melan-A negative melanoma target cell line Na8 was used as a positive and negative control, in absence or presence of the native Melan-A peptide (EAAGIGILTV; “EAA”), respectively. Specific lysis of Na8 target cells was not reduced despite the addition of a low avidity clone (Figure 2C). As expected, lysis was observed only in the presence of the peptide.

### The Presence of Low Avidity T Cells Does Not Reduce the Cytokine Production by High Avidity T Cells

**Figure 3:**
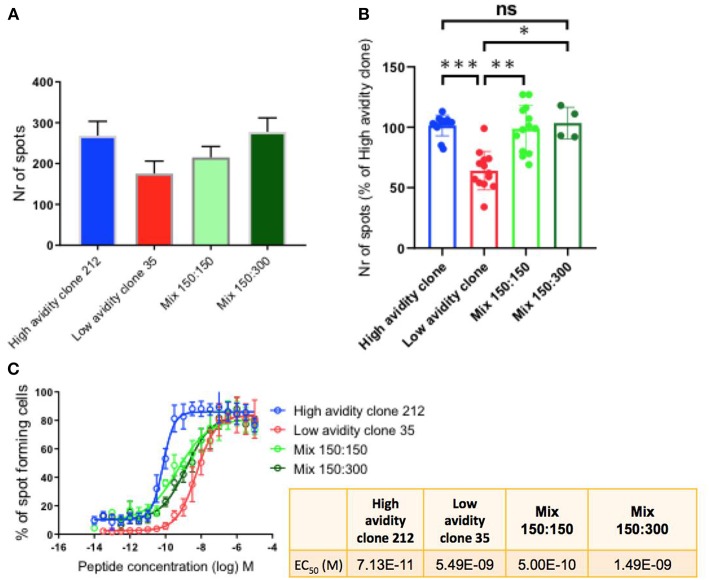
IFN-γ production by T cell clones, alone and in mixed cultures of high and low avidity clones. **(A)** The high avidity clone produced higher spot numbers in comparison to the low avidity clone. After mixing of the clones, plating half of the specific cells of the high avidity clone (150 cells), at ratios 1:1 and 1:2 with the low avidity clone, the absolute total spot numbers were still higher than the single condition of the low avidity clone. **(B)** Compiled data of three experiments showing statistically significant differences, One-way Anova, *P*-values ***<0.001; **<0.01; *<0.05. The T cell clones were analyzed by IFN-γ Elispot assays using T2 cells and 1 μM of Melan-A peptide. **(C)** IFN-γ Elispot assay with titrated peptide concentrations for determining the functional avidity of the clones, alone and as mixtures; the numeric values are shown in the small table.

To determine the cytokine production by the CD8 T cell clones with different functional avidities, we performed IFN-γ Elispot assays, a well-suited method to compare T cell functions of different clones alone, as well as after mixing them in defined ratios (3). The maximum activity was determined when the clones were plated alone (300 cells) and when mixing them in different ratios (1:1 i.e., 150:150; and 1:2 i.e.,150:300; high:low avidity clone). We observed that the high avidity clone 212 produced higher numbers of spots in comparison with the low avidity clone. Similar to the findings in killing assays, the presence of the low avidity clone 35 in the mixtures did not reduce the function of the high avidity clone (Figure 3A). Similar results were found with two different mixtures, namely with the ratios of 150:150 and 150:300 of high:low avidity clones (Figure 3A). Figure 3B shows the compiled results of three experiments, revealing that the differences were statistically significant between the low avidity clone and the other conditions, but no differences between the high avidity clones and the mixtures. The avidity of the clones used was re-verified by IFN-γ Elispot peptide titration assays, confirming the avidity difference between high and low avidity clones (Figure 3C and Table 1). As described previously (3), the mixes of a high avidity clone with a low avidity clone, at a 1:1 ratio (high: low), gave an intermediate EC_50_ value_._ Mixing the clones at 1:2 (150 high: 300 low), the avidity was still higher as compared to the low avidity clone alone.

### The Presence of Low Avidity T Cells Does Not Reduce the Anti-tumor Efficacy of High Avidity T Cells *in vivo*

**Figure 4:**
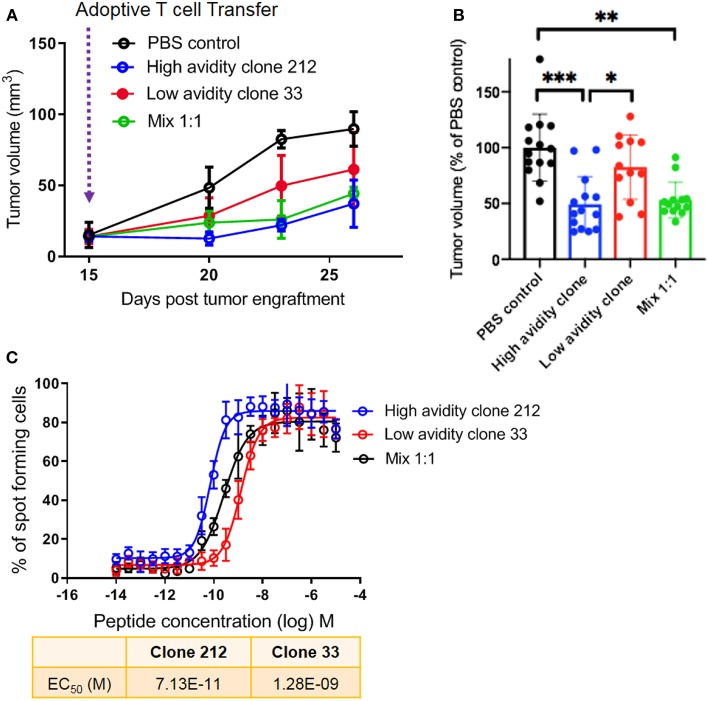
*In vivo* protective capacity of T cell clones in humanized mice. **(A)** 6 to 8 weeks old NSG-A2 mice (groups of 5 mice) were injected subcutaneously on the right flank with 2 × 10^6^ human Me275 melanoma cells. Once the Me275 tumors became palpable at around D23 post tumor engraftment, 1 × 10^6^ of a high avidity T cell clone (blue), a low avidity T cell clone (red) or a 1:1 mixture of the two clones (green) were injected intravenously in the tail vein. The results are from one of three representative experiments. **(B)** Average ± standard deviation, and statistical comparisons (One-way Anova) of tumor volumes at the end of three repeat experiments, substantiating the results in **(A)**. *P-*values ***<0.001; **<0.01; *<0.05. **(C)** IFN-γ Elispot data from the clones used *in vivo*, showing the two distinct functional avidities.

Following these *in vitro* experiments, we aimed to assess the *in vivo* protective capacity of high and low avidity CD8 T cells. We used the model of immunodeficient NSG-HLA-A2 mice that express a human HLA-A2 transgene (further referred to as NSG-A2 mice) to perform adoptive T cell therapy using our clones with defined avidity. Our aim was to assess whether the presence of lower avidity T cells may inhibit the capacity of high avidity T cells to inhibit melanoma growth *in vivo*. We adoptively transferred mixtures of a low avidity T cell clone with a high avidity T cell clone. Interestingly, melanoma growth was similar in these mice as compared to mice transferred with only a single T cell clone with high avidity (Figure 4A). Tumor growth was more rapid in the mice that did not receive T cells, and in mice that received only the low avidity T cells. This experiment was repeated three times, each with similar results, showing significantly reduced tumor growth in animals treated with the high avidity clone and the mixture of high plus low avidity clones, but not the low avidity clone (Figure 4B). Figure 4C displays the IFN-γ Elispot peptide titration curves, showing the functional avidity of the clones used for adoptive transfer to NSG-A2 mice. Together, our results demonstrate that high avidity T cells could inhibit melanoma growth *in vivo*, without reduction of the therapeutic effect in the presence of low avidity T cells. We conclude that the anti-tumor effect of adoptively transferred T cells is not hampered by the co-presence of T cells with lower avidity.

## Discussion

Antigen-specific T cell interactions play central roles in immunity against microbes and cancers. The qualitative and quantitative analysis of functional avidity of individual T cell clonotypes has been important in understanding the cellular immune response in health and disease (25). The sensitivity of a T cell to antigen is influenced by multiple factors: the affinity of the TCR-peptide-MHC interaction, and the engagement of multiple other receptors on T cells and the density of these receptors on the T cell surface within the T cell synapse (4, 26, 27).

Protection from infectious disease is more powerful if the T cell response includes high avidity clones (28). Nevertheless, T cell responses usually also include clones with lower avidity. A too strong selection of particular (high avidity) clones may result in too drastic narrowing of the CD8 T cell repertoire that would render the T cell response more vulnerable. Stochastic recruitment/expansion seems more likely to maintain T cell diversity, shown to be beneficial for instance in virus control (29–31). Also in anti-tumoral T cell responses, generating and maintaining a relatively large number of clonotypes may be more powerful for both safeguarding the flexibility of the repertoire and ensuring an effective immune response.

Our results revealed that low avidity CD8 T cells do not inhibit the function of high avidity CD8 T cells in melanoma. For both cytotoxicity and IFN-γ production, we did not find evidence for inhibition or competition of low avidity T cells that would reduce the interaction of high avidity T cells with peptide-MHC complexes on the surface of tumor cells. Our findings are in contrast to a previous report that low avidity T cells inhibited the activity of high avidity T cells specific for tumor antigens (32). There is increasing evidence that low avidity T cells do not hinder their high avidity counterparts. A study by Zehn et al. showed that even elevated numbers of low avidity memory T cells did not inhibit the response of high avidity T cells (33). For our *in vivo* analysis we used the model of immunodeficient NSG-A2 mice to perform adoptive T cell therapy using our clones with defined avidity. Our results show that also *in vivo*, the anti-tumor effect of high avidity T cells is not hampered by the co-presence of T cells with lower avidity.

While our *in vitro* experiments were limited to the interactions of T cells with tumor cells, our *in vivo* analysis involved also the many other components of the complexity of a complete *in vivo* immune response, including human HLA-A2 expressing antigen-presenting cells. However, humanized mice have the limitation that certain molecular interactions may be altered or absent due to species incompatibilities. Therefore, indirect effects may have been missed, e.g., through modification of antigen presenting cells. A second limitation of our study lies in the fact that using T cell clones is a reductionist approach. *In vivo* T cell responses are polyclonal, i.e., they usually involve more than two clonotypes per epitope specificity. We made attempts to overcome this limitation by studying clones representing multiple clonotypes, in total comprising four high and four low avidity clonotypes (Table 1). We have previously shown that the natural *in vivo* polyclonal responses are relatively well-represented by these clones that were derived from those responses (3). Nevertheless, it should be taken into consideration that our experiments only partially but not fully dissect the complexity of natural polyclonal T cell competition.

High avidity T cells that react to tumor/self-proteins are deleted in the thymus during T cell development, leaving predominantly T cells with lower avidity for recognizing tumor antigens. T cells are also regulated by peripheral tolerance mechanisms, which further reduces the anti-tumor T cell response (34). The avidity of neo-antigen specific T cells may also be preferentially low, possibly because neo-antigens (closely) resemble self-antigens that drive tolerance induction of high avidity T cells. These considerations strengthen the notion that low avidity T cells are abundant and are often generated in immune responses, particularly in cancer patients. Negative impacts of low avidity T cells to the immune response would have unfavorable consequences on immunity. One could postulate that low avidity T cells help to downregulate immune responses, thus could act similarly as regulatory T cells. However, such a model where low avidity T cells have immune regulatory roles would require that these cells are controlled by mechanisms that are distinct to the ones that control high avidity T cells. To our knowledge there is no evidence for the existence of such mechanisms.

It is obviously desirable that vaccines and immunotherapy preferentially activate high avidity T cells, which are more powerful than low avidity T cells. In parallel, a number of strategies are being developed to improve the function of low avidity T cells so that they may be used prophylactically or therapeutically against cancer. Such strategies exploit the binding properties of T cells to tumors both antigen-specifically and non-specifically (35).

Melanoma tumors are often enriched in melanoma-reactive TILs of both high and low avidity, whereby Melan-A-specific T cells are frequently detectable among the melanoma-reactive TILs in HLA-A2 positive patients (36). TILs are regularly used for adoptive T cell therapy which can lead to clinical responses in >50% of melanoma patients thus representing a potentially powerful immunotherapy for metastatic cancer patients. Some strategies select TILs for adoptive transfer, whereas others do not select but rather use the entire TIL population for treating the melanoma patients. Selection does not appear to have a significant impact on the clinical response rate, although this subject requires further studies (37–41).

We conclude that low avidity T cells are likely present in most immune responses and may not hamper their high avidity counterparts. Further studies are necessary that carefully analyze the protective power of T cells with defined functional avidity, with the aim to clarify whether there are exceptions, i.e., whether some low avidity T cells can indeed by harmful for patients in need for protection against cancer.

## Materials and Methods

### Patients

Twenty-nine HLA-A^*^0201–positive patients with stage III/IV metastatic melanoma received series of monthly subcutaneous vaccinations with 0.1 mg Melan-A/MART-1_26−35_ peptide and 0.5 mg CpG 7909/PF-3512676 (Pfizer and Coley Pharmaceutical Group), emulsified in IFA (Montanide ISA-51; Seppic) in a phase I clinical trial (LUD 00-018, ClinicalTrials.gov number NCT00112229, registered May 31, 2005). Eligibility criteria and study design were previously described (42). The trial was conducted according to the relevant regulatory standards, upon approval by Swissmedic (the Swiss agency for therapeutic products), the Protocol Review Committee of the Ludwig Institute for Cancer Research New York and the Ethical Committee for Clinical Research of the Faculty of Medicine of the University of Lausanne. The latter also approved the experimental protocols and the use of PBMC from healthy volunteers. Patients were enrolled upon written informed consent. All methods were carried out in accordance with relevant guidelines and regulations.

### Generation of CD8 T Cell Clones

Blood withdrawal and handling, were performed as previously described (3). Briefly, CD8+ cells were purified using MS columns, loaded with a maximal number of 10 × 10^6^ PBMCs labeled cells, using the MiniMACS™ separators attached to the MACS MultiStand Magnet (Miltenyi). For generating T cell clones, antigen-specific T cells were sorted directly *ex vivo* with fluorescent MHC/peptide tetramers and CD8 mAb, using a FACS Aria cell sorter (BD Biosciences). Sorted T cells were cloned by plating at concentrations of respectively, 0.5 and 1 cell per well, in Terasaki plates, and stimulated with 1 × 10^6^/ml irradiated allogeneic PBMCs (feeder cells), PHA (1 μg/ml), and IL-2 (150 U/ml).

### Chromium Release Cytotoxicity Assay

Chromium release assays were performed using radioactive 51-chromium. For experiments with titrated peptides, 51-chromium-labeled TAP2/2-deficient T2 cells (HLA-A^*^0201+) were pulsed with serial dilutions from 10^−6^ M to 10^−13^ M of Melan-A EAAGIGILTV peptide. For the assessment of tumor cell killing, 1000 Me290 melanoma cells were 51-chromium-labeled and used as target cells. A high avidity CD8 T cell clone alone, a low avidity clone alone, and a 1:1 mix of them (half the amount from each) were tested side-by-side in each experiment. The effector to melanoma cell ratios were 30:1, 10:1, 3:1, 1:1. In the mixtures, the ratio of high to low avidity clones was always 1 to 1. For experiments with variable low:high avidity T cell ratios shown in Figure 2, 1,000 target cells were plated in every well. Different numbers of low avidity T cells were pre-incubated for 1.5 h with Me290 melanoma cells. The high avidity T cells were added in the following cell numbers (ratios) of low avidity clone:high avidity clone, at 80.000:10.000 (8:1), 40.000:10.000 (4:1), 20.000:10.000 (2:1), 10.000:10.000 (1:1), 5.000:10.000 (0.5:1), and incubated for further 3 h to determine specific lysis. Control conditions were performed with the Melan-A negative melanoma cell line Na8, with and without the addition of synthetic Melan-A peptide (“EAA”; EAAGIGILTV). After 4 h incubation, supernatants were analyzed in a TopCount NXT benchtop microplate scintillation and luminescence counter. To determine spontaneous and the total 51-chromium release, medium or 1 M HCL was added in 4 wells with target cells, respectively. The percentage of specific lysis was calculated as 100 × (experimental – spontaneous release)/(total – spontaneous release). All cytotoxicity assay conditions were tested in duplicates, the average of the two values is shown in the figures and was used for data compilation.

### IFN-γ Elispot Assay

Twenty-five microliter of 35% ethanol/well was put in 96 well PVDF plates (MSIPS4510, Millipore) and incubated at room temperature (RT) for 30 s. The wells were emptied by flicking the plate over a sink and gently tapping on absorbent paper. The plates were thoroughly washed 3x with 100 μl 1X PBS per well. Hundred microliter of diluted capture antibody (100 μl into 10 ml 1X PBS) was added to every well and the plates were covered and incubated at 4°C overnight. The wells were washed as previously, once with 100 μl 1X PBS. Hundred microliter of culture medium with 10% serum was added to every well and the plates were incubated at RT for 2 h. The wells were washed with 100 μl 1X PBS, and 300 antigen-specific CD8 T cells in 50 μl of RPMI with 8% Human serum were plated with 50 μl of 20'000 T2 cells per well and the addition of the native Melan-A peptide (EAA, stock 1 mg/ml) at the indicated concentrations, in a total volume of 200 μl per well. The plates were incubated at 37°C in a CO_2_ incubator for 20 h. The next day the plates were incubated with 100 μl of 0.05% PBS-Tween solution (wash buffer) per well at 4°C for 10 min and subsequently 3x with wash buffer. Hundred microliter of diluted detection antibody (100 μl into 10 ml Dilution buffer) was added to every well and incubated at RT for 1 h 30 min. The plates were washed 3x with 100 μl of wash buffer and 100 μl of diluted Streptavidin –AP conjugate was added to every well. After an incubation of 1 h at RT, the plates were washed 3x with wash buffer and 3x with distilled water (both sides of the membrane, peeling off the plate bottom). Hundred microliter of BCIP/NBT buffer was added to every well and the plates were incubated for 5–15 min, monitoring spot formation visually throughout the incubation period for sufficient color development. The wells were emptied and both sides of the membrane were rinsed 3x with distilled water. The frequency of the resulting colored spots corresponding to the cytokine producing cells was determined using the Elispot Bioreader 5000. The data were analyzed with Prism software as previously described (3).

### Mice and Adoptive Transfer of T Cell Clones

The *in vivo* experiments were performed with immunodeficient mice (NSG) transgenic for HLA.A2 [NOD.Cg-PrkdcscidIl2rgtm1WjlTg(HLA-A2.1)1Enge/SzJ mice], referred to as NSG-A2 mice in the text. The mice were purchased from The Jackson Laboratory (Bar Harbor, ME) and then bred in-house. All mice were maintained in specific pathogen free conditions, given autoclaved food and water, and housed in individually ventilated racks in the research animal facilities of the Ludwig Cancer Research Center. Six to eight weeks old NSG-A2 mice (groups of five mice) were injected subcutaneously on the right flank with 2 × 10^6^ human Me275 melanoma cells. Once the Me275 tumors became palpable at around D23 post tumor engraftment, 1 × 10^6^ of a high avidity T cell clone, a low avidity T cell clone, or a 1:1 mixture of the two clones were injected intravenously in the tail vein (100 μl PBS) of mice. 45.000 U IL-2 was injected subcutaneously for the first 2 days. The tumor length and width were measured every 2 days, using a caliper, and the tumor volume was calculated using the formula V = (L × W × W)/2, where V is tumor volume, W is the tumor width and L the tumor length. All animal experiments were conducted in accordance with guidelines of the Animal Care and Use Committee of the Swiss Law and under animal protocols approved by the Veterinary Service of the Canton Vaud.

## Data Availability

All datasets generated for this study are included in the manuscript/supplementary files.

## Ethics Statement

The studies involving human participants were reviewed and approved by Ethical Committee for Clinical Research of the Faculty of Medicine of the University of Lausanne. The patients/participants provided their written informed consent to participate in this study. The animal study was reviewed and approved by Committee of the Swiss Law for Animal Care and Use, University of Lausanne, Switzerland.

## Author Contributions

KI, DV, and DS: conception and design. KI, OR, and AS: acquisition of data. KI, DV, and DS: analysis and interpretation of data. KI, HM-E, DV, and DS: writing, review, and revision of manuscript. KI, PB, and DV: technical and material support. PB, DV, and DS: study supervision.

### Conflict of Interest Statement

The authors declare that the research was conducted in the absence of any commercial or financial relationships that could be construed as a potential conflict of interest.
